# Fibroblast Activation Protein (FAP) as a Serum Biomarker for Fibrotic Ovarian Aging: A Clinical Validation Study Based on Translational Transcriptomic Targets

**DOI:** 10.3390/ijms26167807

**Published:** 2025-08-13

**Authors:** Hyun Joo Lee, Yunju Jo, Shibo Wei, Eun Hee Yu, Sul Lee, Dongryeol Ryu, Jong Kil Joo

**Affiliations:** 1 Department of Obstetrics and Gynecology, Pusan National University School of Medicine, Pusan National University Hospital Biomedical Research Institute, Busan 49241, Republic of Korea; atouchof@pusan.ac.kr (H.J.L.);; 2 Department of Biomedical Science and Engineering, Gwangju Institute of Science and Technology (GIST), Gwangju 61005, Republic of Korea

**Keywords:** ovarian fibrosis, ovarian reserve, AMH, fibroblast activation protein, biomarker, reproductive aging

## Abstract

Chronological age is an imprecise proxy for reproductive capacity, necessitating biomarkers that reflect the underlying pathophysiology of the ovary. Fibrotic remodeling of the ovarian stroma is a key hallmark of biological ovarian aging, yet it cannot be assessed by current clinical tools. This study aimed to identify and validate a novel serum biomarker for fibrotic ovarian aging by applying supervised machine learning (ML) to human ovarian transcriptomic data. Transcriptomic data from the Genotype-Tissue Expression (GTEx) database were analyzed using ML algorithms to identify candidate genes predictive of ovarian aging, and finally, fibroblast activation protein (FAP) and collectin-11 (COLEC11) were selected for clinical validation. In a cross-sectional study, serum levels of FAP and COLEC11, along with key hormonal indices, were measured in two nested patient cohorts, and their associations with ovarian reserve and clinical parameters were analyzed. Serum FAP levels did not correlate with age but showed a strong inverse correlation with anti-Müllerian hormone (AMH) (r = −0.61, *p* = 0.001), a finding accentuated in women with decreased ovarian reserve (DOR). While COLEC11 correlated with age, it failed to differentiate DOR status. FAP levels were independent of central hormonal regulation, consistent with preclinical fibrotic models. Circulating FAP reflects age-independent, fibrotic ovarian aging, offering stromal-specific information not captured by conventional hormonal markers. This study provides the first clinical validation of FAP as a biomarker for ovarian stromal aging, holding potential for improved reproductive risk assessment.

## 1. Introduction

In the current era of increasing maternal age and declining birth rates across the globe, timely and accurate assessment of reproductive potential has become a public health priority [1,2]. Reproductive aging in women is a complex biological process defined by a progressive decline in both the quantity and quality of oocytes, which ultimately determines fertility lifespan; such decline is primarily driven by the depletion of the primordial follicle pool through atresia, a process reflected clinically as a decrease in ovarian reserve (OR) [3]. While decreased ovarian reserve (DOR) is an expected part of natural aging, a growing subset of women of reproductive age experience accelerated ovarian aging, presenting with DOR despite regular menstruation [1]. This discrepancy highlights the significant limitations of using chronological age as a proxy for biological ovarian health and underscores the unmet need for biomarkers that can precisely reflect the underlying pathophysiology of the aging ovary [1,4].

Anti-Müllerian hormone (AMH), a glycoprotein produced by the granulosa cells of preantral and small antral follicles, is currently the most widely used biomarker for quantifying ovarian reserve [5,6]. While its utility in reflecting the size of the follicular pool is well-established, AMH levels are subject to considerable interindividual and intra-cycle variability [7,8]. More importantly, AMH provides little to no information about the health of the ovarian stroma—an increasingly recognized determinant of the follicular microenvironment and overall reproductive aging [9]. In particular, stromal health has been understood to play a pivotal role in supporting folliculogenesis, regulating sufficient ovarian vascularization, and maintaining the biochemical milieu necessary for oocyte competence, making its assessment essential for a more complete evaluation of ovarian aging [9,10]. The stromal compartment undergoes profound age-related remodeling, characterized by fibroblast activation, excessive extracellular matrix (ECM) deposition, and progressive tissue stiffening, a condition termed fibrosis [4,10]. Indeed, a recent comprehensive review highlighted that ovarian stromal fibrosis is not merely a hallmark of aging but could be suggested as a primary cause of ovarian dysfunction; in the ovary, ECM components, such as collagen, increase tissue stiffness, which physically impedes follicular growth and disrupts the essential biomechanical signaling required for follicle–stroma crosstalk [10]. Also, pathologically elevated levels of profibrotic factors, particularly transforming growth factor (TGF)-β, can lead directly to follicular dysplasia and anovulation [10]. Furthermore, fibrosis is associated with reduced interstitial vascularity, which likely impairs the nutrient supply to developing follicles [10]. This process is often driven by a state of chronic, low-grade inflammation, where the sustained elevation of cytokines such as interleukin (IL)-6 and IL-8 can further promote fiber formation and create a hostile microenvironment for oocyte maturation. Consequently, such fibrotic state disrupts crucial follicle–stroma crosstalk, impairs ovarian vascularization, and creates a pro-inflammatory microenvironment that can directly compromise oocyte quality and follicular development [4,10].

Recent preclinical studies have solidified that this fibrotic remodeling is a central feature of ovarian aging [9,10]. A key mediator implicated in this process is fibroblast activation protein (FAP), a serine protease selectively expressed by activated fibroblasts in tissues undergoing active remodeling, such as tumors and fibrotic organs [10]. Unlike static structural fibrosis markers, FAP is enzymatically active and directly involved in ECM degradation and remodeling, thereby reflecting ongoing fibrotic activity rather than a terminal state [10,11]. In the ovary, FAP has been shown to contribute to ECM degradation and remodeling, thereby disrupting the delicate ovarian niche and impairing folliculogenesis [10,11]. While there is accumulating evidence for ovarian fibrosis as a core mechanism of reproductive decline, a non-invasive serum biomarker to monitor this stromal pathology has not been established. This represents a critical blind spot in clinical practice, hindering the early detection of accelerated ovarian aging and the stratification of patients for fertility preservation or targeted therapies.

To address this gap, the current study employed a machine learning-based transcriptomic analysis of ovarian tissue from the Genotype-Tissue Expression (GTEx) dataset to identify candidate genes associated with fibrotic aging signatures, as our group has previously demonstrated the utility of integrative bioinformatic approaches for biomarker discovery in other complex gynecological diseases, such as endometriosis [12]. Such a high-dimensional, unbiased approach is well-suited for isolating biologically relevant features from complex aging-related gene expression profiles. Herein, we report the translational validation of FAP and collectin-11 (COLEC11)—two candidates identified through this approach—in a clinically annotated patient cohort. By linking molecular markers of stromal aging with the established endocrine indices of ovarian reserve, this study aims to advance the development of fibrosis-specific biomarkers that extend beyond follicular enumeration, laying the groundwork for their integration into clinical paradigms for reproductive risk assessment.

## 2. Results

### 2.1. ML Identification of Possible Candidates with Divergent Age-Related Expression in Ovarian Tissue

To identify novel biomarkers, this study first applied an ML pipeline to the ovarian transcriptome dataset from the GTEx project (*n* = 180). Such an unbiased approach identified several high-confidence candidate genes predictive of ovarian aging. FAP was prioritized for validation as its tissue-level mRNA expression showed a significant positive correlation with chronological age, with Spearman’s *Rho* = 0.17, *p* = 0.021, as stated in Supplementary Figure S1A. In contrast, another candidate, COLEC11, did not show a clear linear correlation with age, as stated in Supplementary Figure S1B; however, it was consistently ranked as a top predictor for classifying age groups in the ML models. Thus, given its high feature importance in the models and its known biological role in fibrosis, COLEC11 was selected as a compelling second candidate for clinical validation [13].

### 2.2. Circulating FAP Specifically Reflects Ovarian Reserve in Real-World Samples

**Figure 1:**
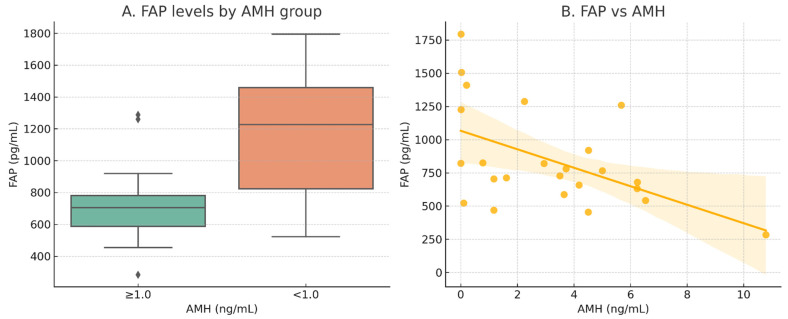
Serum fibroblast activation protein (FAP) distribution across biological ovarian aging metrics: (**A**) comparison of FAP levels between women with anti-Müllerian hormone (AMH) ≥ 1.0 ng/mL and <1.0 ng/mL (DOR group); error bars represent interquartile range (*p* = 0.045); (**B**) scatter plot of FAP versus AMH levels, with linear regression line (orange) and shaded 95% confidence interval (r = −0.61, *p* = 0.001).

This study validated the two candidate biomarkers in a clinical cohort of 72 women, and the primary correlation analyses are summarized in Table 1. In contrast to its tissue expression pattern, circulating FAP levels showed no correlation with chronological age (*r* = 0.089, *p* = 0.458). Conversely, circulating COLEC11 levels strongly correlated with age (*r* = 0.415, *p* < 0.001), a finding inconsistent with its tissue expression but typical of many systemic aging markers, as described in Table 2. In the hormone-integrated subcohort (*n* = 24), serum FAP levels exhibited a significant inverse correlation with serum AMH levels (*r* = −0.61, *p* = 0.001), as presented in Figure 1A,B. Importantly, FAP levels were independent of the centrally regulated gonadotropins, FSH and LH. Further multivariate regression analysis confirmed that AMH was the only significant independent predictor of serum FAP levels, as presented in Supplementary Tables S1–S3.

## 3. Discussion

The central finding of this study is the identification and clinical validation of circulating FAP as a novel biomarker that specifically reflects ovarian reserve, independent of chronological age. The current translational approach, which began with an unbiased ML-based screening of ovarian transcriptomes, pinpointed FAP as a key candidate associated with aging. Subsequent validation in the clinical cohort, however, revealed a crucial distinction: while another candidate, COLEC11, correlated with chronological age, only serum FAP showed a strong and significant inverse relationship with AMH. Furthermore, the lack of correlation between FAP and centrally regulated gonadotropins such as FSH and LH suggests its role as an intrinsic marker of ovarian pathophysiology, rather than a participant in the systemic endocrine feedback loop. These collective findings establish a strong foundation for evaluating FAP as a clinically relevant indicator of biological ovarian aging.

While chronological age remains a cornerstone of fertility assessment, its inability to capture the vast individual variations in the rate of reproductive decline is a major clinical limitation [14,15,16]. In the current study, FAP expressions in ovarian tissue samples was initially observed to have a positive correlation with age, probably representing a gradual, subclinical increase in stromal stress or activation that accumulates over decades; however, the strong inverse correlation between serum FAP protein and AMH suggests that a significant elevation of FAP into circulation occurs only when this stromal remodeling process becomes pathologically robust enough to disrupt the follicular microenvironment and accelerate the depletion of the ovarian reserve [10,11,17]. Therefore, circulating FAP may not be a marker of accumulated time, but rather a dynamic, real-time indicator of an ongoing, detrimental fibrotic process within the ovary.

The findings of the current study could also provide preclinical evidence implicating ovarian stromal fibrosis as a key driver of reproductive decline. For decades, the clinical assessment of ovarian aging has been predominantly follicle-centric; recently, a growing body of research recognizes the ovarian stroma not as a passive scaffold, but as an active, dynamic compartment essential for orchestrating folliculogenesis and determining oocyte competence [18,19]. Pathological aging is associated with profound stromal remodeling, leading to excessive ECM deposition, increased tissue stiffness, and a state of chronic, low-grade inflammation, or “inflammaging” [20,21,22]. Mechanistically, FAP functions as an active serine protease that cleaves fibrillar and basement–membrane collagens, including types I, III, and IV, as well as other ECM substrates, thereby amplifying matrix turnover and cortical stiffening. FAP expression is upregulated by profibrotic cytokines such as TGF-β, IL-1β, and TNF-α, and can occur in both membrane-bound and soluble—“shed”—forms that remodel the microenvironment at a distance [23]. ECM stiffening then propagates integrin–FAK–RhoA signaling and nuclear translocation of YAP/TAZ, the “Hippo effector,” altering granulosa/stromal transcriptional programs that govern follicle growth and oocyte competence; concomitantly, TGF-β/SMAD activity sustains myofibroblast differentiation, collagen cross-linking, and a feed-forward fibrotic loop [11,24,25]. At the tissue level, collagen-rich, anisotropic stromal architecture and age-related ovarian stiffness correlate with impaired perifollicular perfusion and aberrant angiogenic signaling, disrupting granulosa–oocyte crosstalk and accelerating atresia [26]. Importantly, antifibrotic interventions that deplete stromal collagen restore ovulation and embryo developmental competence in both aged and obese mouse models, supporting the causal role of fibrosis [10,25,26]. FAP is a well-established and highly specific mediator of such processes, known for its pivotal role in tissue remodeling in cancer stroma and fibrotic diseases of the liver and lung [23,24,25,26,27,28]. By linking this fundamental fibrosis-associated protein to a clinical marker of ovarian reserve for the first time, the current study suggests that circulating FAP may offer a much-needed, non-invasive window into the health of the ovarian stromal microenvironment, a domain previously accessible only through invasive histological analysis.

The clarity of this biological window provided by FAP is further underscored when contrasted with the results for COLEC11. Despite also being identified as a fibrosis-related candidate, COLEC11 exhibited a completely different profile in our clinical validation. Its circulating levels showed no association with ovarian reserve but instead demonstrated a significant positive correlation with chronological age. This pattern suggests that circulating COLEC11 may simply be a general marker of systemic, temporal aging, one that is more closely related to the general aging process and its associated inflammation [13,29]. Future studies involving larger and more diverse patient cohorts are warranted to fully elucidate the role of COLEC11 in the context of female reproductive health.

The potential clinical utility of a validated stromal biomarker like FAP in the current study could substantially address several key unmet needs in reproductive medicine [30]. Current research emphasizes that established markers such as AMH, while useful, are poor predictors of individual reproductive potential, sometimes discordant with other ovarian reserve markers and especially in natural pregnancy cohorts, and should not be misconstrued as definitive “fertility tests” [31,32,33,34,35]. This limitation has fueled an intense search for novel biomarkers that reflect different aspects of ovarian biology. For instance, recent elegant studies have utilized advanced techniques to identify new potential regulators of ovarian aging linked to transcriptional programs, such as FOXP1, or mitochondrial dysfunction, like NSUN4 [30,36]. While these studies provide invaluable mechanistic insights, the current study adds a distinct and crucial dimension by offering the first clinical validation of a circulating biomarker for ovarian stromal fibrosis—a critical component of ovarian aging that has been largely inaccessible in clinical practice [10,11,17]. Furthermore, the current study proposes that circulating FAP is not a replacement for AMH, but a crucial complementary partner, providing qualitatively different information on ovarian health. The clinical utility of existing ovarian reserve markers is primarily confined to estimating oocyte quantity, while they remain poor predictors of oocyte quality or overall reproductive potential [6,32]. The health of the ovarian stroma is an increasingly recognized determinant of the follicular microenvironment and, by extension, oocyte competence [4,9,10]. Therefore, the discordance between a marker of follicular quantity, possibly represented by AMH, and a marker of stromal pathology, possibly represented by FAP, could provide a more nuanced assessment of a patient’s true biological ovarian age, supposedly in DOR with young age and/or women with premature ovarian insufficiency (POI). Such an added dimension—a non-invasive window into stromal health—could empower more precise risk stratification for accelerated reproductive decline, thereby enabling more personalized and timely counseling for fertility preservation. In the context of assisted reproductive technology (ART), integrating a stromal health marker could refine the prognostic landscape for patients, offering a more complete picture beyond the predicted oocyte yield. Looking further ahead, as FAP is an enzyme directly involved in tissue remodeling, it represents not just a biomarker but a potential therapeutic target [9,11,25]. The ability to monitor an active fibrotic process may open the door to future interventions with antifibrotic agents aimed at preserving or improving the ovarian niche [10,25].

The current study poses a notable strength in its robust translational design, which progressed from an unbiased, data-driven bioinformatic discovery phase to rigorous validation in a clinically annotated cohort. To the best of our knowledge, this is the first study to propose and clinically validate a circulating biomarker with a direct link to ovarian stromal pathophysiology. However, several limitations must be acknowledged. As a foundational, proof-of-concept study, the cross-sectional design allows us to demonstrate a strong association, yet it cannot establish causality or determine the predictive value of FAP for future fertility outcomes. Moreover, while statistically significant, our findings are derived from a modest-sized cohort at a single tertiary hospital, which may limit their generalizability. Previous research states that the prevalence of POI in the general population is estimated at 1–3.7% among women under 40 years of age, and that up to 10% of patients undergoing ART may present with DOR related to infertility [37,38]. Nonetheless, recruiting participants who meet strict inclusion and exclusion criteria and provide high-quality biospecimens remains a considerable logistical challenge, particularly outside an ART setting. These limitations, while important, do not diminish the novelty of our findings but rather, along with the current controlled design and strict phenotyping of the study cohort, provide a clear framework for the future research directions required to integrate FAP into clinical practice and translate it into a standard clinical tool. The most critical next step may be conducting large-scale, prospective longitudinal studies; following a cohort of women over time is essential to determine whether an elevated FAP level can predict the future rate of AMH decline and, ultimately, clinical outcomes such as time to pregnancy or ART success. Such studies will also be crucial for establishing the clinically meaningful cut-off values for risk stratification. Furthermore, it is imperative to validate the utility of FAP in specific patient populations at high risk for accelerated ovarian aging, particularly women with POI, who represent the most extreme phenotype of this condition. Investigating FAP in this group would provide a powerful test of its biological relevance. Still, in rare cases of POI due to inactivating FSH receptor (FSHR) mutations, where AMH can be paradoxically high, FAP levels may not be elevated initially [39]. This scenario highlights that FAP should be viewed not as a universal predictor for all forms of POI, but rather as a biomarker specifically predictive of ovarian dysfunction driven by stromal fibrosis. In mechanistically distinct forms of POI, such as those due to inactivating FSHR mutations, the primary defect lies in gonadotropin signaling, and stromal architecture may initially remain intact, possibly resulting in normal FAP levels despite impaired folliculogenesis. Recognizing this distinction is critical for refining the clinical utility of FAP, as such an approach can allow for targeted application in conditions where fibrotic remodeling is a primary or early driver of ovarian decline, thereby increasing its predictive value within the appropriate patient subset. Finally, further mechanistic studies using animal models or organoid cultures are warranted to elucidate the precise cellular source of circulating FAP and understand how its enzymatic activity directly contributes to follicular attrition.

To summarize, the current study elevates FAP from a bioinformatically identified candidate to a clinically validated biomarker of biological ovarian aging. By demonstrating a robust, age-independent correlation with ovarian reserve, our findings establish circulating FAP as the first non-invasive proxy for the health of the ovarian stromal microenvironment. This work proposes a conceptual shift in reproductive aging assessment: moving beyond the quantitative enumeration of follicles, as provided by AMH, to a more functional and mechanistic evaluation of the underlying stromal pathology that drives follicular decline. The ability to monitor this detrimental fibrotic process not only provides a new tool for risk stratification but also opens a new frontier for developing more precise diagnostics and, ultimately, novel therapeutic strategies to preserve female fertility.

## 4. Materials and Methods

### 4.1. Bioinformatic Identification of Ovarian Aging Biomarker Candidates

#### 4.1.1. Data Acquisition and Preprocessing

To identify the candidate biomarkers of accelerated ovarian aging, transcriptomic data and corresponding clinical metadata of normal human ovarian tissues were retrieved from the GTEx project portal (v8; dbGaP accession number phs000424.v8.p2), and the analysis was performed using R (version 4.4.2; R Foundation for Statistical Computing, Vienna, Austria). Gene-level expression data, quantified as transcripts per million (TPM), were log^2^-transformed. To minimize batch effects across samples, quantile normalization was applied using the preprocessCore R package (Bioconductor, Seattle, WA, USA).

#### 4.1.2. Supervised Machine Learning (ML) and Feature Selection

To identify genes predictive of biological ovarian aging, this study implemented supervised machine learning (ML) algorithms to classify ovarian tissues based on age. Samples were categorized into a “younger” group with ages between 20 and 39 years, and an “older” group with ages of 40 years and above. Two distinct ensemble learning models, Random Forest, using the randomForest package, and XGBoost, using the xgboost package (version 2.0.3), were applied. The predictive importance of each gene—“feature”—was evaluated using the mean decrease in Gini index for Random Forest and SHapley Additive exPlanations Additive exPlanations (SHAP) values for XGBoost. A consensus list of top-ranking genes robustly identified by both models was generated for further analysis.

#### 4.1.3. Pathway Enrichment Analysis

To prioritize the candidates related to stromal pathophysiology, the consensus gene list was subjected to pathway enrichment analysis. Gene Ontology (GO) and Kyoto Encyclopedia of Genes and Genomes (KEGG) analyses were performed using the JEPETTO plugin (version 1.3.1) of Cytoscape (version 3.8.2). Genes significantly enriched in pathways related to “extracellular matrix”, “ovarian failure”, and “ovarian reserve” were prioritized. Through this pipeline, FAP and COLEC11 were selected as high-confidence candidates for subsequent clinical validation.

### 4.2. Clinical Validation Study Design

#### Cohort Recruitment

A cross-sectional clinical validation study was conducted at a single, tertiary, university hospital setting. The study included a primary cohort (*n* = 72) of reproductive-aged and menopausal women undergoing gynecological evaluation and a subcohort (*n* = 24) with concurrent hormonal measurements. Within the reproductive-aged cohort with ages between 18 and 45 years, a subgroup with biochemically low ovarian reserve, hereafter referred to as the DOR group for analytical purposes, was defined as having a serum AMH < 1.0 ng/mL (*n* = 12). This threshold was chosen to enable an exploratory subanalysis on the relationship between FAP and a biochemically confirmed state of low follicular reserve, as in previous research [31,40].

### 4.3. Laboratory Measurements

#### 4.3.1. Serum Biomarker Quantification

Serum levels of FAP and COLEC11 were quantified in duplicate using commercial sandwich enzyme-linked immunosorbent assay (ELISA) kits according to the manufacturers’ protocols. Specifically, FAP levels were measured using the Human FAP DuoSet ELISA Kit (R&D Systems, Minneapolis, MN, USA). COLEC11 levels were measured using the Human CL-K1/COLEC11 ELISA Kit (Novus Biologicals, Centennial, CO, USA). For both assays, standard curves were generated using a four-parameter logistic (4-PL) curve-fit, and sample concentrations were calculated from the curve. The inter- and intra-assay coefficients of variation (CVs) for the measurements performed in our laboratory were maintained below 10%.

#### 4.3.2. Hormonal Assays

Peripheral blood samples for hormonal analysis were collected during the follicular phase of the menstrual cycle. Serum AMH was measured using the MIS/AMH ELISA Kit (Beckman Coulter, High Wycombe, Buckinghamshire, UK), which has manufacturer-stated intra- and inter-assay CVs of less than 12.3% and 14.2%, respectively. Serum follicle-stimulating hormone (FSH) and luteinizing hormone (LH) levels were assessed using ImmuChem™ Coated Tube Radioimmunoassay (RIA) Kits (MP Biomedicals, Santa Ana, CA, USA).

### 4.4. Statistical Analyses

All statistical procedures were performed using R, version 4.4.2. Pearson correlation was used to assess relationships between biomarker levels and continuous clinical variables. Group comparisons were made using independent *t*-tests. Multivariable linear regression models were used to assess independent associations. A *p*-value < 0.05 was considered statistically significant.

## 5. Conclusions

In conclusion, this study successfully identified and validated circulating FAP as a novel, non-invasive biomarker for biological ovarian aging. By demonstrating a specific, age-independent correlation with the decline in ovarian reserve, FAP provides a unique window into the pathophysiology of the ovarian stroma, a critical component of reproductive health that has been largely inaccessible in clinical practice. The introduction of FAP into the clinical armamentarium, not as a replacement but as a complement to AMH, has the potential to shift the paradigm of fertility assessment from a quantitative measure of the follicle pool to a more comprehensive evaluation that includes the health of the microenvironment. This work paves the way for more precise risk stratification, personalized fertility counseling, and proactive reproductive management for women worldwide.

## Figures and Tables

**Table 1:** Summary of key correlations between biomarkers and primary clinical parameters. Analysis was performed using Pearson correlation analyses. FAP, fibroblast activation protein; COLEC11, collectin-11; AMH, anti-Müllerian hormone; FSH, follicle-stimulating hormone; LH, luteinizing hormone.

Biomarker	Correlated Parameter	Cohort	Correlation Coefficient (r)	*p*-Value
FAP	Chronological Age	Overall (*n* = 72)	0.089	0.458
	AMH	Subcohort (*n* = 24)	−0.517	0.01
	FSH	Subcohort (*n* = 24)	0.147	0.492
	LH	Subcohort (*n* = 24)	−0.012	0.957
COLEC11	Chronological Age	Overall (*n* = 72)	0.415	<0.001
	AMH	Subcohort (*n* = 24)	−0.258	0.223

**Table 2:** Comparison of circulating biomarker levels by age group (<45 versus ≥45 years) using independent *t*-tests: (**A**) overall cohort (*n* = 72); (**B**) hormone-integrated subcohort (*n* = 24). Data are presented as mean ± standard deviation (SD). Comparisons were made between women aged <45 years and ≥45 years for each biomarker (COLEC11 and FAP). Statistically significant *p*-values are shown in bold (*p* < 0.05). Abbreviations: COLEC11, collectin-11; FAP, fibroblast activation protein; WBC, white blood cell count; CA125, cancer antigen 125; FSH, follicle-stimulating hormone; LH, luteinizing hormone; AMH, anti-Müllerian hormone.

A								
	Overall (*n* = 72)		<45 years (*n* = 46)		≥45 years (*n* = 26)			
Variables	Mean	SD	Mean	SD	Mean	SD	t	*p*-Value
**COLEC11**	197.26	51.80	183.25	38.48	222.06	62.87	−2.860	0.007
**FAP**	859.20	353.16	798.12	269.29	967.27	452.40	−1.740	0.091
**B**								
	**Overall** **(*n* = 24)**		**<45 years** **(*n* = 18)**		**≥45 years** **(*n* = 6)**			
**Variables**	**Mean**	**SD**	**Mean**	**SD**	**Mean**	**SD**	**t**	***p*** **-Value**
**COLEC11**	169.06	35.66	163.31	30.64	186.29	46.71	−1.127	0.300
**FAP**	849.63	374.26	760.72	297.38	1116.36	479.40	−1.711	0.135

## Data Availability

The raw data supporting the conclusions of this article will be made available by the authors on request.

## Supplementary Materials

The following supporting information can be downloaded at: https://www.mdpi.com/article/10.3390/ijms26167807/s1.

## References

[1] Chon S.J., Umair Z., Yoon M.S. (2021). Premature Ovarian Insufficiency: Past, Present, and Future. Front. Cell Dev. Biol..

[2] Jiao X., Meng T., Zhai Y., Zhao L., Luo W., Liu P., Qin Y. (2021). Ovarian Reserve Markers in Premature Ovarian Insufficiency: Within Different Clinical Stages and Different Etiologies. Front. Endocrinol..

[3] Cavalcante M.B., Sampaio O.G.M., Câmara F.E.A., Schneider A., de Ávila B.M., Prosczek J., Masternak M.M., Campos A.R. (2023). Ovarian aging in humans: Potential strategies for extending reproductive lifespan. GeroScience.

[4] Zeng Y., Wang C., Yang C., Shan X., Meng X.Q., Zhang M. (2024). Unveiling the role of chronic inflammation in ovarian aging: Insights into mechanisms and clinical implications. Hum. Reprod..

[5] Jamil Z., Fatima S.S., Ahmed K., Malik R. (2016). Anti-Mullerian Hormone: Above and Beyond Conventional Ovarian Reserve Markers. Dis. Markers.

[6] Karaviti E., Karaviti D., Kani E.R., Chatziandreou E., Paschou S.A., Psaltopoulou T., Kalantaridou S., Lambrinoudaki I. (2025). The role of anti-Müllerian hormone: Insights into ovarian reserve, primary ovarian insufficiency, and menopause prediction. Endocrine.

[7] Jeong H.G., Kim S.K., Lee J.R., Jee B.C. (2022). Correlation of oocyte number with serum anti-Müllerian hormone levels measured by either Access or Elecsys in fresh in vitro fertilization cycles. Clin. Exp. Reprod. Med..

[8] Kim J.H., Kim H.O., Lee S.Y., Park E.A., Choi K.H., Kang K., Yu E.J., Koong M.K., Lee K.A. (2025). Serum miR-329-3p as a potential biomarker for poor ovarian response in an in vitro fertilization. Clin. Exp. Reprod. Med..

[9] Isola J.V.V., Ocañas S.R., Hubbart C.R., Ko S., Mondal S.A., Hense J.D., Carter H.N.C., Schneider A., Kovats S., Alberola-Ila J. (2024). A single-cell atlas of the aging mouse ovary. Nat. Aging.

[10] Umehara T., Winstanley Y.E., Andreas E., Morimoto A., Williams E.J., Smith K.M., Carroll J., Febbraio M.A., Shimada M., Russell D.L. (2022). Female reproductive life span is extended by targeted removal of fibrotic collagen from the mouse ovary. Sci. Adv..

[11] Gu M., Wang Y., Yu Y. (2024). Ovarian fibrosis: Molecular mechanisms and potential therapeutic targets. J. Ovarian Res..

[12] Bae S.J., Jo Y., Cho M.K., Jin J.S., Kim J.Y., Shim J., Kim Y.H., Park J.-K., Ryu D., Lee H.J. (2022). Identification and analysis of novel endometriosis biomarkers via integrative bioinformatics. Front. Endocrinol..

[13] Wu W., Liu C., Farrar C.A., Ma L., Dong X., Sacks S.H., Li K., Zhou W. (2018). Collectin-11 Promotes the Development of Renal Tubulointerstitial Fibrosis. J. Am. Soc. Nephrol..

[14] Davis S.R., Pinkerton J., Santoro N., Simoncini T. (2023). Menopause—Biology, consequences, supportive care, and therapeutic options. Cell.

[15] Jin C., Wang X., Yang J., Kim S., Hudgins A.D., Gamliel A., Pei M., Contreras D., Devos M., Guo Q. (2025). Molecular and genetic insights into human ovarian aging from single-nuclei multi-omics analyses. Nat. Aging.

[16] Steiner A.Z., Jukic A.M.Z. (2016). Impact of female age and nulligravidity on fecundity in an older reproductive age cohort. Fertil. Steril..

[17] Ma J., Wang L., Yang D., Luo J., Gao J., Wang J., Guo H., Li J., Wang F., Wu J. (2024). Chronic stress causes ovarian fibrosis to impair female fertility in mice. Cell. Signal..

[18] Camaioni A., Ucci M.A., Campagnolo L., De Felici M., Klinger F.G. (2022). The process of ovarian aging: It is not just about oocytes and granulosa cells. J. Assist. Reprod. Genet..

[19] Shen L., Liu J., Luo A., Wang S. (2023). The stromal microenvironment and ovarian aging: Mechanisms and therapeutic opportunities. J. Ovarian Res..

[20] López-Otín C., Blasco M.A., Partridge L., Serrano M., Kroemer G. (2023). Hallmarks of aging: An expanding universe. Cell.

[21] García-Domínguez M. (2025). Pathological and Inflammatory Consequences of Aging. Biomolecules.

[22] Franceschi C., Garagnani P., Parini P., Giuliani C., Santoro A. (2018). Inflammaging: A new immune–metabolic viewpoint for age-related diseases. Nat. Rev. Endocrinol..

[23] Basalova N., Alexandrushkina N., Grigorieva O., Kulebyakina M., Efimenko A. (2023). Fibroblast Activation Protein Alpha (FAPα) in Fibrosis: Beyond a Perspective Marker for Activated Stromal Cells?. Biomolecules.

[24] Piccolo S., Dupont S., Cordenonsi M. (2014). The biology of YAP/TAZ: Hippo signaling and beyond. Physiol. Rev..

[25] Amargant F., Magalhaes C., Pritchard M.T., Duncan F.E. (2025). Systemic low-dose anti-fibrotic treatment attenuates ovarian aging in the mouse. GeroScience.

[26] Ouni E., Peaucelle A., Haas K.T., Van Kerk O., Dolmans M.-M., Tuuri T., Otala M., Amorim C.A. (2021). A blueprint of the topology and mechanics of the human ovary for next-generation bioengineering and diagnosis. Nat. Commun..

[27] Yang A.T., Kim Y.O., Yan X.Z., Abe H., Aslam M., Park K.S., Zhao X.Y., Jia J.D., Klein T., You H. (2023). Fibroblast Activation Protein Activates Macrophages and Promotes Parenchymal Liver Inflammation and Fibrosis. Cell. Mol. Gastroenterol. Hepatol..

[28] Lavis P., Garabet A., Cardozo A.K., Bondue B. (2024). The fibroblast activation protein alpha as a biomarker of pulmonary fibrosis. Front. Med..

[29] Howard M., Farrar C.A., Sacks S.H. (2018). Structural and functional diversity of collectins and ficolins and their relationship to disease. Semin. Immunopathol..

[30] Hao J., Liu L., Chang B., Zhao Y., Lai Y., Tian C., Xu H., Wang H., Ji L., Yang J. (2025). Blood-detected mitochondrial biomarker NSUN4: A potential indicator of ovarian aging. Exp. Gerontol..

[31] Salemi F., Jambarsang S., Kheirkhah A., Salehi-Abargouei A., Ahmadnia Z., Hosseini H.A., Lotfi M., Amer S. (2024). The best ovarian reserve marker to predict ovarian response following controlled ovarian hyperstimulation: A systematic review and meta-analysis. Syst. Rev..

[32] Baker K.M., FernandezCriado R., Eaton J.L., Mensah V.A. (2025). The Clinical Utility of Measures of Ovarian Reserve. Obstet. Gynecol. Surv..

[33] Lee H.J., Noh H.K., Joo J.K. (2022). Comparison of ART outcome in patients with poor ovarian response according to POSEIDON criteria. Sci. Rep..

[34] Peigné M., Bernard V., Dijols L., Creux H., Robin G., Hocké C., Grynberg M., Dewailly D., Sonigo C. (2023). Using serum anti-Müllerian hormone levels to predict the chance of live birth after spontaneous or assisted conception: A systematic review and meta-analysis. Hum. Reprod..

[35] Yu E.H., Lee H.J., Joo J.K., Na Y.J. (2024). Which Ovarian Reserve Marker is More Reliable in IVF Patients with AMH and AFC Discordance?. Clin. Exp. Obstet. Gynecol..

[36] Wu M., Tang W., Chen Y., Xue L., Dai J., Li Y., Zhu X., Wu C., Xiong J., Zhang J. (2024). Spatiotemporal transcriptomic changes of human ovarian aging and the regulatory role of FOXP1. Nat. Aging.

[37] Golezar S., Keshavarz Z., Ramezani Tehrani F., Ebadi A., Zayeri F., Golezar M.H. (2022). Primary ovarian insufficiency quality of life scale (POIQOLS): Development and psychometric properties. BMC Women’s Health.

[38] Golezar S., Ramezani Tehrani F., Khazaei S., Ebadi A., Keshavarz Z. (2019). The global prevalence of primary ovarian insufficiency and early menopause: A meta-analysis. Climacteric.

[39] Cooper O.O., Quint E.H., Smith Y.R., Dendrinos M.L. FSH receptor variant: An unusual cause of secondary amenorrhea. J. Pediatr. Adolesc. Gynecol..

[40] Iwase A., Osuka S., Goto M., Murase T., Nakamura T., Takikawa S., Manabe S., Kikkawa F. (2023). Anti-Müllerian hormone for screening, diagnosis, evaluation and prediction of functional ovarian reserve: A clinical perspective. J. Obstet. Gynaecol. Res..

