# Supplementary material for: Fibroblast Activation Protein (FAP) as a Serum Biomarker for Fibrotic Ovarian Aging: A Clinical Validation Study Based on Translational Transcriptomic Targets
Source: Int J Mol Sci. 2025 Aug 13;26(16):7807. doi: 10.3390/ijms26167807 (PMC12386806; doi:10.3390/ijms26167807)

SUPPLEMENTARY FIGURE LEGENDS

Supplementary Figure S1. Divergent expression patterns of FAP and COLEC11 in human ovarian tissue in relation to chronological age. Bioinformatic analysis of the GTEx project ovarian transcriptome dataset. Box plots show mRNA expression levels (log_2[TMM+1]) across different age decades.

A. FAP tissue expression demonstrated a significant positive correlation with age (Spearman's Rho=0.17, *p*=0.021).

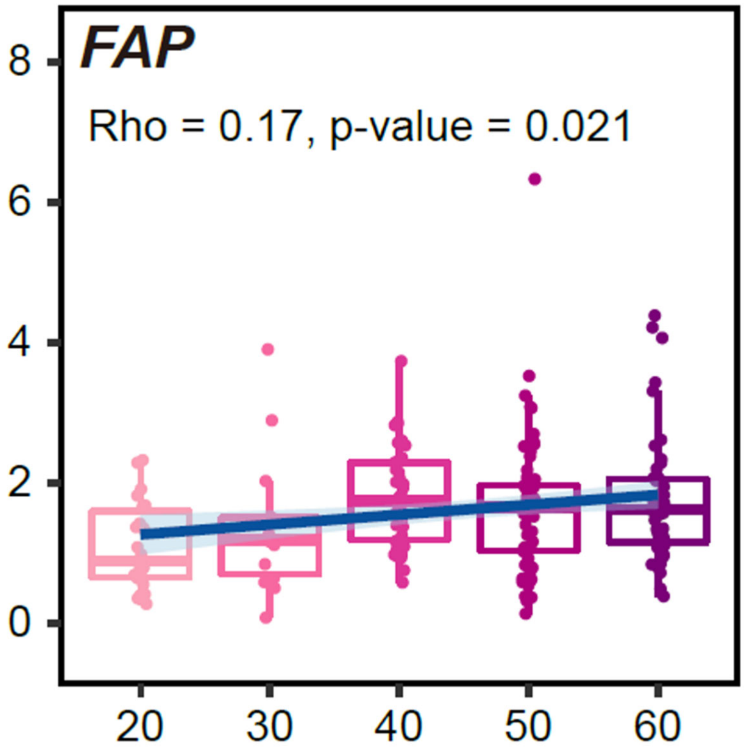

B. COLEC11 tissue expression showed no clear or significant linear correlation with chronological age.

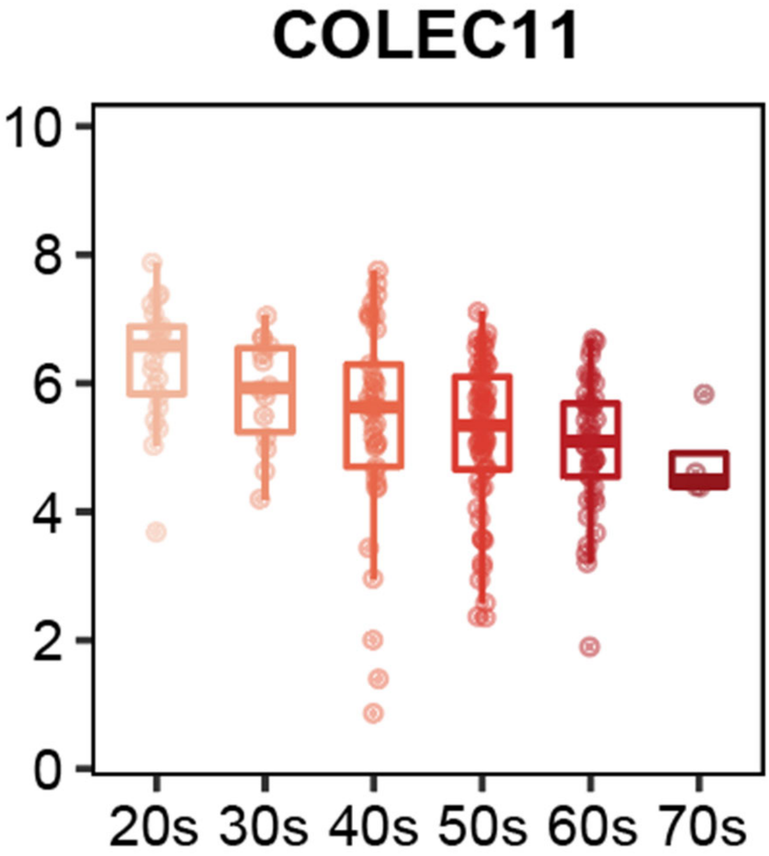

Supplement: Supplementary file 1 [file ijms-26-07807-s001.zip › ijms-3793437-Suppkementary Figure.pdf]
