# Supplementary material for: Fibroblast Activation Protein (FAP) as a Serum Biomarker for Fibrotic Ovarian Aging: A Clinical Validation Study Based on Translational Transcriptomic Targets
Source: Int J Mol Sci. 2025 Aug 13;26(16):7807. doi: 10.3390/ijms26167807 (PMC12386806; doi:10.3390/ijms26167807)
Supplement: Supplementary file 1 [file ijms-26-07807-s001.zip › ijms-3793437-Suppkementary Tables.pdf]

Supplementary Table S1. Pearson correlation coefficients between circulating biomarkers and clinical parameters. A. Overall cohort (n=72). B. Hormone-integrated subcohort (n=24). Correlation analyses were performed using the Pearson method. Values are shown as correlation coefficient (r) with p-values in parentheses. Statistically significant correlations (*p*<0.05) are shown in bold. Abbreviations: collectin-11, COLEC11; fibroblast activation protein, FAP; white blood cell count, WBC; cancer antigen 125, CA125; follicle-stimulating hormone, FSH; luteinizing hormone, LH; anti-Mullerian hormone, AMH.

A.

	**Age**	**COLEC11**	**FAP**	**WBC**	**CA125**
**Age**	1				
**COLEC11**	**0.415 (*p*<.001)**	1			
**FAP**	0.089 (*p*=0.458)	-0.075 (*p*=0.531)	1		
**WBC**	0.073 (*p*=0.542)	0.074 (*p*=0.535)	0.019 (*p*=0.874)	1	
**CA125**	-0.123 (*p*=0.368)	-0.025 (*p*=0.857)	-0.028 (*p*=0.836)	-0.148 (*p*=0.275)	1

B.

	**Age**	**COLEC11**	**FAP**	**WBC**	**CA125**	**FSH**	**LH**	**AMH**
**Age**	1							
**COLEC11**	0.237 (*p*=0.264)	1						
**FAP**	0.361 (*p*=0.083)	0.28 (*p*=0.186)	1					
**WBC**	-0.038 (*p*=0.860)	0.205 (*p*=0.336)	0.322 (*p*=0.125)	1				
**CA125**	0.196 (*p*=0.358)	**0.521 (*p*=0.009)**	-0.016 (*p*=0.942)	0.22 (*p*=0.302)	1			
**FSH**	**0.595 (*p*=0.002)**	0.084 (*p*=0.696)	0.147 (*p*=0.492)	-0.105 (*p*=0.626)	-0.207 (*p*=0.333)	1		
**LH**	0.108 (*p*=0.617)	0.07 (*p*=0.745)	-0.012 (*p*=0.957)	-0.009 (*p*=0.967)	-0.239 (*p*=0.261)	**0.613 (*p*=0.001)**	1	
**AMH**	**-0.72 (*p*<.001)**	-0.258 (*p*=0.223)	**-0.517 (*p*=0.010)**	-0.122 (*p*=0.569)	-0.054 (*p*=0.801)	-0.36 (*p*=0.084)	-0.104 (*p*=0.628)	1

Supplementary Table S2. Univariate and multivariate linear regression models evaluating associations of clinical variables with serum COLEC11 and FAP concentrations in overall cohort. Each biomarker was regressed on clinical and demographic predictors using univariate and multivariate linear models. Coefficients (B), standard errors (SE), t-values, and *p*-values are reported. "Group" refers to comorbid benign gynecological diagnostic category, with the reference group being women with normal ovarian findings. Non-ovary-related group included uterine fibroid or endometrial polyp cases, and the ovary-related group included ovarian cystadenoma and mature teratoma cases. Statistically significant associations (p<0.05) are shown in bold. Abbreviations: collectin-11, COLEC11; fibroblast activation protein, FAP; white blood cell count, WBC.

	**COLEC11**								**FAP**							
	Univariate analysis				Multivariate analysis				Univariate analysis				Multivariate analysis			
	B	SE	t	*p*-value	B	SE	t	*p*-value	B	SE	t	*p*-value	B	SE	t	*p*-value
**Age**	1.693	.444	3.815	**.000**	1.449	.843	1.718	.091	2.471	3.312	.746	.458	10.120	6.219	1.627	.109
**WBC**	1.252	2.010	.623	.535	-.630	2.199	-.287	.775	2.190	13.742	.159	.874	5.931	16.218	.366	.716
**Group=normal**																
**Group=ovary-related**	31.868	16.121	1.977	.052	32.122	18.372	1.748	.085	32.153	116.256	.277	.783	24.154	135.504	.178	.859
**Group=non-overy-related**	45.774	13.410	3.413	**.001**	39.711	15.652	2.537	**.014**	158.712	96.701	1.641	.105	159.965	115.439	1.386	.171
**Parity=Yes**	30.183	11.799	2.558	**.013**	7.622	15.684	.486	.629	11.649	84.114	.138	.890	-64.328	115.680	-.556	.580
**Menopause=Yes**	31.667	16.056	1.972	.053	-14.193	24.438	-.581	.563	-105.647	111.762	-.945	.348	-265.402	180.240	-1.472	.146

Supplementary Table S3. Univariate and multivariate linear regression models evaluating associations of clinical variables with serum COLEC11 and FAP concentrations in the hormone-integrated sub-cohort. Each biomarker was regressed on clinical, demographic and hormonal predictors using univariate and multivariate linear models. Coefficients (B), standard errors (SE), t-values, and *p*-values are reported. "Group" refers to comorbid benign gynecological diagnostic category, with the reference group being women with normal ovarian findings. Non-ovary-related group included uterine fibroid or endometrial polyp cases, and the ovary-related group included ovarian cystadenoma and mature teratoma cases. Statistically significant associations ($p<0.05$) are shown in bold. Abbreviations: collectin-11, COLEC11; fibroblast activation protein, FAP; white blood cell count, WBC; cancer antigen 125, CA125; follicle-stimulating hormone, FSH; luteinizing hormone, LH; anti-Mullerian hormone, AMH.

	**COLEC**								**FAP**							
	Univariate analysis				Multivariate analysis				Univariate analysis				Multivariate analysis			
	B	SE	t	*p*-value	B	SE	t	*p*-value	B	SE	t	*p*-value	B	SE	t	*p*-value
**Age**	.941	.821	1.146	.264	-2.142	1.666	-1.286	.225	15.040	8.274	1.818	.083	-8.745	19.638	-.445	.665
**WBC**	4.220	4.292	.983	.336	-3.827	6.493	-.589	.568	69.481	43.586	1.594	.125	-95.352	76.525	-1.246	.239
**Group=normal**																
**Group=ovary-related**	-3.085	22.619	-.136	.893	-44.380	38.638	-1.149	.275	260.703	267.412	.975	.341	519.457	455.414	1.141	.278
**Group=non-overy-related**	54.152	16.774	3.228	**.004**	6.013	39.229	.153	.881	370.879	198.318	1.870	.075	360.465	462.383	.780	.452
**Parity=Yes**	8.347	14.777	.565	.578	-5.882	22.839	-.258	.802	116.294	154.247	.754	.459	369.610	269.191	1.373	.197
**Menopause=Yes**	.778	26.926	.029	.977	-302.288	311.334	-.971	.352	190.903	279.679	.683	.502	3933.830	3669.586	1.072	.307

**CA125**	2.789	.973	2.865	**.009**	1.927	2.814	.685	.508	-.881	11.971	-.074	.942	-42.565	33.167	-1.283	.226
**FSH**	.190	.479	.396	.696	6.265	5.970	1.050	.316	3.486	4.987	.699	.492	-72.269	70.361	-1.027	.326
**LH**	.475	1.444	.329	.745	-.313	2.538	-.123	.904	-.833	15.197	-.055	.957	10.928	29.912	.365	.722
**AMH**	-3.309	2.640	-1.253	.223	-4.300	3.977	-1.081	.303	-69.584	24.551	-2.834	**.010**	-25.303	46.878	-.540	.600
